# Supplementary material for: How Well Do Dogs Cope with Air Travel? An Owner-Reported Survey Study
Source: Animals (Basel). 2023 Oct 4;13(19):3093. doi: 10.3390/ani13193093 (PMC10571552; doi:10.3390/ani13193093)
Supplement: Supplementary file 1 [file animals-13-03093-s001.zip › animals-2652372-supplementary.pdf]

How well did your dog cope with air travel?

1. Thank you for participating in this survey and research project

Instructions for completing this survey:
This survey is intended for dog owners who have transported a dog by air in the past 12 months. We are interested in all types of flights, whether a short/domestic or a long/international flight, all responses are welcome!
If you have transported more than one dog by air in the last 12 months, please choose the dog whose name comes first in the alphabet and answer the survey questions for this dog.

Dear dog owner,
My name is Dr Katrin Jahn and I am conducting a research project on how well dogs cope with air travel.

The aim of this survey is to gather more information and data about pet air travel and to improve pet wellbeing and welfare during air transportation.

This research project is part of my residency training to become a specialist in Veterinary Behaviour Medicine and is being supervised by Dr Theresa DePorter, Dr Kersti Seksel and Dr Jacqui Ley.

This survey is completely voluntary and you may stop the survey or participation in the project at any time if you wish.
The survey is completely anonymous and de-identified.

The results of this survey will be analyzed and published in an international journal as well as presented at international conferences.

By completing this questionnaire, you acknowledge your understanding that you cannot be identified via the manuscript and that the authors cannot identify you in any way.
By completing this questionnaire you consent to your information being collected, stored, and published in the future. If at any point you wish for your data to be withdrawn, please email the below email address.

Thank you so much for your participation.
Contact information: drkatrin@germanvet.ae

Supervisors:
Dr Theresa DePorter, BSc, DVM, MRCVS, DECAWBM, DACVB
Dr Kersti Seksel, BVSc (Hons) MRCVS MA (Hons) FACVSc DACVB DECAWBM
Dr Jacqui Ley, BVSc (Hons), FANZCVS (Veterinary Behaviour), PhD DECAWBM

How well did your dog cope with air travel?

2. Questions about your dog

1. What is the name of your dog that traveled by air within the last 12 months? (if more than one, pick the name of the dog that comes first alphabetically - all following questions will refer to this dog)

2. How old is your dog now?

- Under 6 months of age
- 6-24 months of age
- Over 2 years - 4 years of age
- Over 4 years - 6 years of age
- Over 6 years - 8 years of age
- Over 8 years - 12 years of age
- Over 12 years of age

3. What gender and neuter status is your dog?

- Male entire (not neutered/castrated)
- Male neutered/castrated
- Female entire (not sterilised/spayed)
- Female sterilised/spayed

4. Do you consider your dog to be a pure breed dog?

- Yes, my dog is a pure breed dog
- No, my dog is a cross breed dog

5. Is your dog a brachycephalic / snub-nosed / short-nosed dog?

- Yes
- No
- Unsure

6. What is your dog's breed?

7. What is your dog's size and weight?

- ◯ A miniature / "teacup" dog (5 kg/11 lbs or under)
- ◯ A small dog (over 5 kg/11 lbs - 10 kg/22 lbs)
- ◯ A medium dog (over 10 kg/22 lbs - 25 kg/55 lbs)
- ◯ A large dog (over 25 kg/55 lbs - 40 kg/88 lbs)
- ◯ A giant dog (over 40 kg/88 lbs)

8. How would you best describe your dog's haircoat? (please check all that apply)

- ☐ Hairless
- ☐ Short hair
- ☐ Medium hair
- ☐ Long hair
- ☐ Dense / thick hair
- ☐ Curly hair
- ☐ Other (please specify)

9. How old was your dog when you acquired him/her?

- ◯ Under 8 weeks of age
- ◯ 8 - 16 weeks of age
- ◯ Over 4 months-24 months of age
- ◯ Over 2 years-4 years of age
- ◯ Over 4 years-6 years of age
- ◯ Over 6 years-8 years of age
- ◯ Over 8 years-12 years of age
- ◯ Over 12 years of age

10. Where did you acquire your dog from?

- ◯ A registered private breeder
- ◯ A mass breeder / puppy mill
- ◯ A shelter / rescue organisation
- ◯ A family member
- ◯ A pet shop
- ◯ Rescued from the street / previous stray animal
- ◯ Other (please specify)

11. For what reason/s do you have this dog? (please check all that apply)

- [ ] Companionship
- [ ] For the children / as part of the family
- [ ] Because of its breed
- [ ] Because of its appearance / aesthetics
- [ ] As a working dog
- [ ] As a guard dog
- [ ] As a sports / competition dog
- [ ] As a service dog
- [ ] As an emotional support dog
- [ ] To rescue the dog / give him/her a good home / do a good deed
- [ ] From a friend or family member who could no longer keep the dog
- [ ] Took on the dog following the passing of a friend / family member
- [ ] Other (please specify)

12. Does your dog have any physical health concerns? (please check all that apply)

- [ ] Osteoarthritis / chronic musculoskeletal pain
- [ ] Skin allergies
- [ ] Food hypersensitivities
- [ ] Gastro-intestinal disease
- [ ] Heart condition
- [ ] Respiratory tract condition
- [ ] Recurring ear infections
- [ ] Kidney disease
- [ ] Liver disease
- [ ] Endocrine disease (diabetes, Cushing's disease, Addison's disease, thyroid disease)
- [ ] Neoplasia / Tumors / Cancer
- [ ] None
- [ ] Other (please specify)

13. Does your dog have any behaviour or mental / emotional ill health problems? (please check all that apply)

- [ ] Separation Anxiety
- [ ] Noise phobia / Noise sensitivity
- [ ] Anxiety Disorder
- [ ] Fears / phobias of specific triggers (eg. cars, traffic, being approached by unfamiliar people)
- [ ] Aggressive / reactive behaviours
- [ ] Compulsive Disorder (eg. tail chasing, fly snapping, shadow chasing, compulsive licking of self, others or objects)
- [ ] None
- [ ] Other (please specify)

14. Is your dog taking any medication/s?

- ( ) Yes
- ( ) No

15. **If you answered yes to the previous question**, please state what medication/s your dog is taking:

16. What level of training has your dog received throughout its life? (please check all that apply)

- [ ] Attended puppy class
- [ ] Basic obedience training (positive reinforcement)
- [ ] Basic obedience training (balanced)
- [ ] Training with the use of an e-stim / electric shock collar
- [ ] Advanced training (eg. agility, flyball, obedience competition, advanced search)
- [ ] Training at home
- [ ] Training and certification as service dog
- [ ] My dog has not received any training
- [ ] Unknown
- [ ] Other (please specify)

17. How many times has this dog traveled by air in his/her life (including the most recent travel event)

- Never
- 1 time
- 2 times
- 3 times
- 4 times
- 5 times
- 6 - 10 times
- 11 - 20 times
- Over 20 times

3. Questions about you

18. What is your age?

- Under 18
- 18-24
- 25-34
- 35-44
- 45-54
- 55-64
- 65+
- Prefer not to answer

19. What is your gender?

- Female
- Male
- Other
- Prefer not to answer

20. What is the highest level of education you have completed?

- Did not attend school
- Completed primary school / elementary school
- Graduated from high school
- Graduated from college
- Completed an undergraduate degree
- Completed a postgraduate degree
- Completed a certificate, diploma or trade certificate
- Prefer not to answer
- Other (please specify)

21. Which country do you live in?

22. What is your nationality?

23. How stressed were you about the thought of your dog traveling by air?

- ◯ Extremely stressed
- ◯ Very stressed
- ◯ Somewhat stressed
- ◯ Not very stressed
- ◯ Not at all stressed

How well did your dog cope with air travel?

4. Questions about your dog's air travel process and experience

24. When did {{ Q1 }} travel by air (most recent flight) ?

- Over 12 months ago
- Less than 12 months ago
- Less than 6 months ago
- Less than 3 months ago
- Less than 4 weeks ago

25. What was the primary reason for transporting {{ Q1 }} by air?

- Relocation to live in another country
- Holiday / vacation / travel
- This was how I obtained my {{ Q1 }} when he/she was a puppy
- Rehoming my dog to live with someone else
- Adopting a dog from overseas / distance
- Travelling for work with the dog (eg. search and rescue)
- Travelling for my work
- {{ Q1 }} is a service dog and accompanied me on the flight
- {{ Q1 }} is an emotional support animal and accompanied me on the flight
- Other (please specify)

26. **If {{ Q1 }} is an emotional support animal**, did he/she fly in the cabin with you or in the hold of the aircraft?

- In the cabin with me
- In the hold of the aircraft
- {{ Q1 }} is not an emotional support animal

27. **If {{ Q1 }} is an emotional support animal**, and flew in the cabin with you, what was the reason for {{ Q1 }} flying in the cabin with you? (please check all that apply)

- ☐ I needed {{ Q1 }} to fly in the cabin with me to support me
- ☐ I was worried about {{ Q1 }}'s well-being on the flight and thought it would be better if he/she were in the cabin with me
- ☐ It would have been more expensive for {{ Q1 }} to fly in cargo or booked as excess baggage
- ☐ {{ Q1 }} is not an emotional support animal
- ☐ {{ Q1 }} did not fly in the cabin with me
- ☐ I prefer not to answer this question
- ☐ Other (please specify)

28. How old was {{ Q1 }} at the time of air travel (the most recent air travel event)?

- ○ Under 6 months of age
- ○ 6-24 months of age
- ○ Over 2 years - 4 years of age
- ○ Over 4 years - 6 years of age
- ○ Over 6 years - 8 years of age
- ○ Over 8 years - 12 years of age
- ○ Over 12 years of age
- ○ My dog has never travelled by air before

29. Which country did {{ Q1 }}'s flight originate from?

30. Which country did {{ Q1 }} fly to (can be the same country as in the previous question if it was a domestic flight)?

31. Were there any transits or stops during the flight?

- ○ No, it was a direct flight
- ○ Yes, one transit/stop
- ○ Yes, multiple transits/stops

32. At any time during {{ Q1 }}'s trip, was he/she cared for at a dedicated airport animal lounge? (check the answer that best applies to {{ Q1 }}'s trip or all that apply)

- ☐ No, at no point
- ☐ Yes, before the flight
- ☐ Yes, upon arrival
- ☐ Yes, at transit / stopover points
- ☐ I do not know what a dedicated airport animal lounge is or which airports have these
- ☐ I do not know where my dog was cared for after handover and before being reunited with them

33. How long was {{ Q1 }}'s trip in total (including ground travel, the flight and possible boarding or quarantine stays)? (check the option that best applies)

- ○ Short trip (1-6 hours)
- ○ Medium trip (Over 6 hours-10 hours)
- ○ Long trip (Over 10 hours-24 hours)
- ○ Multi-leg trip with multiple stop-overs (Over 24-48 hours)
- ○ Long or multi-leg trip including boarding or quarantine stay/s (Over 48 hours)
- ○ Other (please specify)

34. Did {{ Q1 }} travel in the cabin with you or an accompanying person (flight buddy) or in the hold of the aircraft?

- ○ In the cabin with me on leash
- ○ In the cabin with me in a soft sided carrier
- ○ In the cabin with an accompanying person (flight buddy) on leash
- ○ In the cabin with an accompanying person (flight buddy) in a soft sided carrier
- ○ In the hold of the aircraft
- ○ Other (please specify)

35. Did you use a pet shipping company/agent to help organise {{ Q1 }}'s air travel or did you organise {{ Q1 }}'s air travel yourself?

- ○ I used a pet shipping company for the entire process
- ○ I organised my dog's air travel process myself
- ○ I used a pet shipping agent for some aspects and organised other aspects myself

36. How did you prepare {{ Q1 }} for air travel? (please check all that apply)

- [ ] Familiarisation to the travel crate
- [ ] A comprehensive physical health exam by my veterinarian
- [ ] All necessary vaccinations, blood tests and anti-parasite treatment
- [ ] Microchip placement
- [ ] Pheromone products (eg. Adaptil)
- [ ] Calming supplements
- [ ] Calming diet
- [ ] Anti-anxiety medication prescribed by my veterinarian
- [ ] CBD/Hemp products
- [ ] Aromatherapy
- [ ] None of the above
- [ ] N/A - I did not prepare my dog for travel myself
- [ ] Other (please specify)

37. If you used a medication or supplement prescribed by your veterinarian to prepare {{ Q1 }} for air travel, which medication/s did you use? (please check all that apply) - the names in brackets are brand names of the medications.

- ☐ Acepromazine (ACP)
- ☐ Alprazolam (Xanax)
- ☐ Diazepam (Valium)
- ☐ Trazodone (Desyrel)
- ☐ Dexmedetomindine oromucosal gel (Sileo)
- ☐ Clonidine (Catapres)
- ☐ Gabapentin (Neurontin)
- ☐ Pregabalin (Lyrica)
- ☐ Diphenhydramine (Benadryl)
- ☐ Zylkene
- ☐ Anxitane
- ☐ Composure
- ☐ YuCalm
- ☐ Solliquin
- ☐ Maropitant (Cerenia)
- ☐ None of the above
- ☐ Don't know
- ☐ Other (please specify)

38. How long prior to air travel did you begin preparing {{ Q1 }}?

- ○ 24 hours
- ○ Over 24 hours - 1 week
- ○ Over 1 week - 4 weeks
- ○ Over 1 month - 3 months
- ○ Over 3 months - 6 months
- ○ More than 6 months
- ○ N/A I did not prepare my dog for travel myself

39. Who did you ask for advice when preparing {{ Q1 }} for air travel? (please check all that apply)

- [ ] Veterinarian
- [ ] Pet shipping agent
- [ ] Dog trainer
- [ ] Other pet care professional (groomer, dog walker etc.)
- [ ] Family and friends
- [ ] IPATA or ATA website
- [ ] IATA website
- [ ] Airline / airline website
- [ ] Internet/google searches
- [ ] Social media
- [ ] Read books
- [ ] Own previous experience
- [ ] None of the above
- [ ] Other (please specify)

40. How distressed did you feel {{ Q1 }} was at the following points during his/her journey?

	Extremely distressed	Very distressed	Moderately distressed	Not so distressed	Not at all distressed	N/A
At handover (either to a pet shipper or at cargo desk) or check in before the flight	○	○	○	○	○	○
At the airport and/or during the flight (if traveling in the cabin with you) - please answer N/A if your dog traveled in the aircraft hold	○	○	○	○	○	○
Upon arrival / when handed back to you at the destination	○	○	○	○	○	○

41. What behaviours did {{ Q1 }} show that made you think he/she was distressed at the following points during the journey (if applicable)?

	At handover (either to a pet shipper or at cargo desk) or check-in before flight	At the airport and / or during the flight (if they traveled in the aircraft cabin with you)	Upon arrival or when handed back to you at the destination
Trembling	☐	☐	☐
Panting	☐	☐	☐
Hypersalivating / drooling	☐	☐	☐
Pacing	☐	☐	☐
Pawing	☐	☐	☐
Cowering	☐	☐	☐
Hiding / trying to make themselves small	☐	☐	☐
Growling / showing teeth	☐	☐	☐
Whining / crying	☐	☐	☐
Howling	☐	☐	☐
Barking	☐	☐	☐
Vomiting / diarrhea	☐	☐	☐
Urinating	☐	☐	☐
Tense body	☐	☐	☐
Licking lips	☐	☐	☐
Yawning	☐	☐	☐
Scratching themselves	☐	☐	☐
Shake off	☐	☐	☐
My dog did not show any signs of distress	☐	☐	☐
Other	☐	☐	☐

Other (please specify)

____

42. Did {{ Q1 }} receive medical attention from a veterinarian immediately after air travel?

◯ Yes

◯ No

43. **If you answered yes to the previous question** and {{ Q1 }} received veterinary attention immediately after the flight, what was the reason for this?

- ◯ Routine health exam after air travel
- ◯ To evaluate and treat minor health issues after air travel
- ◯ To evaluate and treat major health issues after air travel
- ◯ To evaluate and treat heat exhaustion or dehydration after air travel
- ◯ To evaluate and treat mental / emotional / behavioural problems after air travel
- ◯ To evaluate and treat already existing physical, mental or emotional / behavioural health problems
- ◯ The visit was unrelated to air travel
- ◯ To get a refill of current medication
- ◯ Other (please specify)

44. How much more stressed than usual was {{ Q1 }} at the following points in time after air travel?

	Extremely more stressed than usual	Much more stressed than usual	Moderately more stressed than usual	Not much more stressed than usual	Not at all more stressed than usual
In the first 48 hours after air travel	◯	◯	◯	◯	◯
In the first 3-7 days after air travel	◯	◯	◯	◯	◯
In days 8-30 after air travel	◯	◯	◯	◯	◯
Over 30 days after air travel	◯	◯	◯	◯	◯

45. What stress behaviours did {{ Q1 }} display at the following times after air travel? (please check all that apply)

	In the first 48 hours after air travel	In the first 3-7 days after air travel	In days 8-30 after air travel	Over 30 days after air travel
Hiding	☐	☐	☐	☐
Less interactive	☐	☐	☐	☐
More clingy	☐	☐	☐	☐
Not eating	☐	☐	☐	☐
Not drinking	☐	☐	☐	☐
Excessive thirst	☐	☐	☐	☐
Ravenous appetite	☐	☐	☐	☐
Changes in toileting behaviour (house soiling)	☐	☐	☐	☐
Diarrhea	☐	☐	☐	☐
Constipation	☐	☐	☐	☐
Unable to settle	☐	☐	☐	☐
Hyperactive	☐	☐	☐	☐
Excessive barking	☐	☐	☐	☐
Sleeping less or poor quality sleep	☐	☐	☐	☐
Sleeping more	☐	☐	☐	☐
Reluctant to go for a walk	☐	☐	☐	☐
Anxious or more anxious	☐	☐	☐	☐
Scared of specific triggers (eg. cars, traffic, being approached by unfamiliar people)	☐	☐	☐	☐
Aggressive (growling, snarling, lunging, biting)	☐	☐	☐	☐
None of the above	☐	☐	☐	☐
Other	☐	☐	☐	☐

Other (please specify)

____

46. Did {{ Q1 }} develop any behaviour problems within 3 months after air travel? (please check all that apply)

- ☐ No
- ☐ Yes, separation anxiety
- ☐ Yes, noise phobias / noise sensitivities
- ☐ Yes, he/she is generally more anxious
- ☐ Yes, he/she is fearful of specific triggers (eg. cars, traffic, being approached by unfamiliar people)
- ☐ Yes, aggressive or reactive behaviours
- ☐ Yes, he/she has developed compulsive behaviours (eg tail chasing, fly snapping, shadow chasing, compulsive licking on self, others or objects)
- ☐ Other (please specify)

47. **If you answered yes to the previous question**, which of the following factors do you think contributed to the development of the behaviour problem/s ?

- ☐ Air travel / the flight
- ☐ Change in physical environment (eg. new home, different lifestyle, different climate)
- ☐ Change in social environment (eg. new people, leaving known people behind, new dogs / animals, leaving known dogs / animals behind)
- ☐ Other (please specify)

48. Did {{ Q1 }} experience a worsening of an already existing behaviour problem within 3 months after air travel? (please check all that apply)

- ☐ No
- ☐ Yes, separation anxiety
- ☐ Yes, noise phobias / noise sensitivities
- ☐ Yes, their anxiety disorder is worse
- ☐ Yes, their fear of specific triggers is worse or more generalised
- ☐ Yes, aggressive or reactive behaviours
- ☐ Yes, compulsive behaviours
- ☐ Other (please specify)

49. **If you answered yes to the previous question**, which of the following factors do you think contributed to the worsening of the behaviour problem/s ?

- ☐ Air travel / the flight
- ☐ Change in physical environment (eg. new home, different lifestyle, different climate)
- ☐ Change in social environment (eg. new people, leaving known people behind, new dogs / animals, leaving known dogs / animals behind)
- ☐ Other (please specify)

50. Did {{ Q1 }} experience the development or worsening of any physical health problems within 3 months after air travel? (please check all that apply)

- ☐ No
- ☐ Yes, osteoarthritis / chronic musculoskeletal pain
- ☐ Yes, skin allergies
- ☐ Yes, food hypersensitivities
- ☐ Yes, gastro-intestinal disease
- ☐ Yes, heart condition
- ☐ Yes, respiratory tract condition
- ☐ Yes, recurring ear infections
- ☐ Yes, kidney disease
- ☐ Yes, liver disease
- ☐ Yes, endocrine disease (diabetes, Cushing's disease, Addison's disease, thyroid disease)
- ☐ Yes, neoplasia, tumors, cancer
- ☐ Other (please specify)

51. **If you answered yes to the previous question**, which of the following factors do you think contributed to the development or worsening of your dog's physical health ?

- ☐ Air travel / the flight
- ☐ Change in physical environment (eg. new home, different lifestyle, different climate)
- ☐ Change in social environment (eg. new people, leaving known people behind, new dogs / animals, leaving known dogs / animals behind)
- ☐ A factor most likely unrelated to air travel or a new environment
- ☐ Other (please specify)
